# Force delivery modification of removable thermoplastic appliances using Hilliard precision thermopliers for tipping an upper central incisor

**DOI:** 10.1007/s00784-022-04560-4

**Published:** 2022-05-31

**Authors:** Bernhard Wiechens, Phillipp Brockmeyer, Teresa Erfurth-Jach, Wolfram Hahn

**Affiliations:** 1 Department of Orthodontics, University Medical Centre Goettingen, University of Goettingen, Robert-Koch-Str. 40, 37075 Goettingen, Germany; 2 Department of Oral and Maxillofacial Surgery, University Medical Centre Goettingen, Robert-Koch-Str. 40, 37075 Goettingen, Germany; 3 Bremen, Germany; 4 Goettingen, Germany

**Keywords:** Removable thermoplastic appliances; Hilliard precision thermopliers; Force delivery; Aligner biomechanics

## Abstract

**Objectives:**

To evaluate the force delivered by removable thermoplastic appliances (RTAs, aligners), altered with Hilliard precision thermopliers, on an upper central incisor to tip it in the palatal and vestibular directions.

**Materials and methods:**

A total of 10 aligners made from Ideal Clear® (polyethylene terephthalate glycol copolyester, PET-G) with a thickness of 1 mm were used in force analysis. Different-sized spot-thermoformed protuberances (bumps) were generated by activating the thermoplier (thin and thick) up to 30°, 60° and 90° in the centre of the palatal and vestibular surfaces of the aligner in 15° steps. The tipping (Fx) and intrusive (Fz) force components were measured on the isolated upper central incisor as part of a standardized resin model, with or without vertical loading by a weight equivalent.

**Results:**

Thermoplier activation at 30°, 60° and 90° resulted in different bump heights. The analysis revealed significantly higher Fx and Fz values with increasing bump heights for every activation step in all cases (*p* < 0.0001, respectively). Overall, the values of the Fx force component were higher than those observed for Fz. Significant differences between the palatal and vestibular tipping procedures were found depending on the resulting force components when the thin thermoplier was used; in contrast, the thick thermoplier resulted in a larger dispersion of the force magnitudes.

**Conclusions:**

Aligners modified with Hilliard precision thermopliers showed altered biomechanical parameters. This approach could be an option for treatment modification.

**Clinical relevance:**

The instrumental examination provided informative results for daily practice, as activation, force dosage and different force values under chewing pressure can be estimated more precisely based on the determined force levels.

## Background

Removable thermoplastic appliances (RTAs or aligners) are used in patients with permanent dentition as a less visible alternative to conventional fixed orthodontic appliances [1–6] or as removable retainers after patients undergo treatment [7, 8]. The choice of material thickness and composition is application-related; this relation has been the subject of numerous clinical studies [9–12]. In general, among the number of recommendations, however, 0.75-mm-thick polyethylene terephthalate glycol copolyester (PET-G) seems to be preferred for active tooth movement, and 1.00-mm-thick PET-G is preferred for retention [10, 13]. In contrast to retention purposes, active tooth movement by aligners generates the force necessary for tooth movement by causing local and full-body deformation of the aligner when it is placed on the tooth row. This deformation occurs due to a fitting discrepancy between the actual and intended tooth positions, which is incorporated into the appliance [14–16]. At present, this procedure has emerged to be the most common and is also suggested to treat distinct malocclusions using 3D printers, in which a further model is created for every tooth position change (step) of at least 1 mm [13]. As an alternative, RTAs can also be used without a primary fitting discrepancy, and thus without multiple subsequent malocclusion models, by activating a single aligner using spot-thermoforming. It must be emphasized in this context that tooth movement with this technique occurs in free space, whereas additional activation surfaces (e.g., power ridges) are also used in the fitting discrepancy technique, for example, to support difficult torque movements [5]. As an extension of the original Essix retainer system (Dentsply-Raintree Essix, Sarasota, FL 34,243, USA) [8], Sheridan et al. added windows and divots to thermoplastic retainers to allow minor tooth movements [17]; this design subsequently developed into an active chairside treatment approach with thermally activated pliers for minor tooth alignment [18, 19]. For this approach, Hilliard precision thermopliers (Dentsply-Raintree Essix, Sarasota, FL 34,243, USA) can be used to create small protuberances on the inner surface of the appliance (bumps) [19]. Space must be created at the position of the tooth target by cutting a window into the RTA or by blocking out the cast before thermoforming [19]. When an aligner previously modified by spot-thermoforming is positioned, the tooth to be treated is exposed to the bump, and therefore, the aligner is not placed completely onto the tooth row. Consequently, the appliance becomes deformed when pressed into the target position, and the resulting force components press the tooth through the bump. Pertinently, different biomechanical studies have described the force delivery of thermoformed aligners during tooth movement [1, 14–16, 20–23]. Especially under the conditions of chewing pressure, the force transmission of RTAs modified by spot-thermoforming has not yet been investigated.

## Materials and methods

**Fig. 1:**
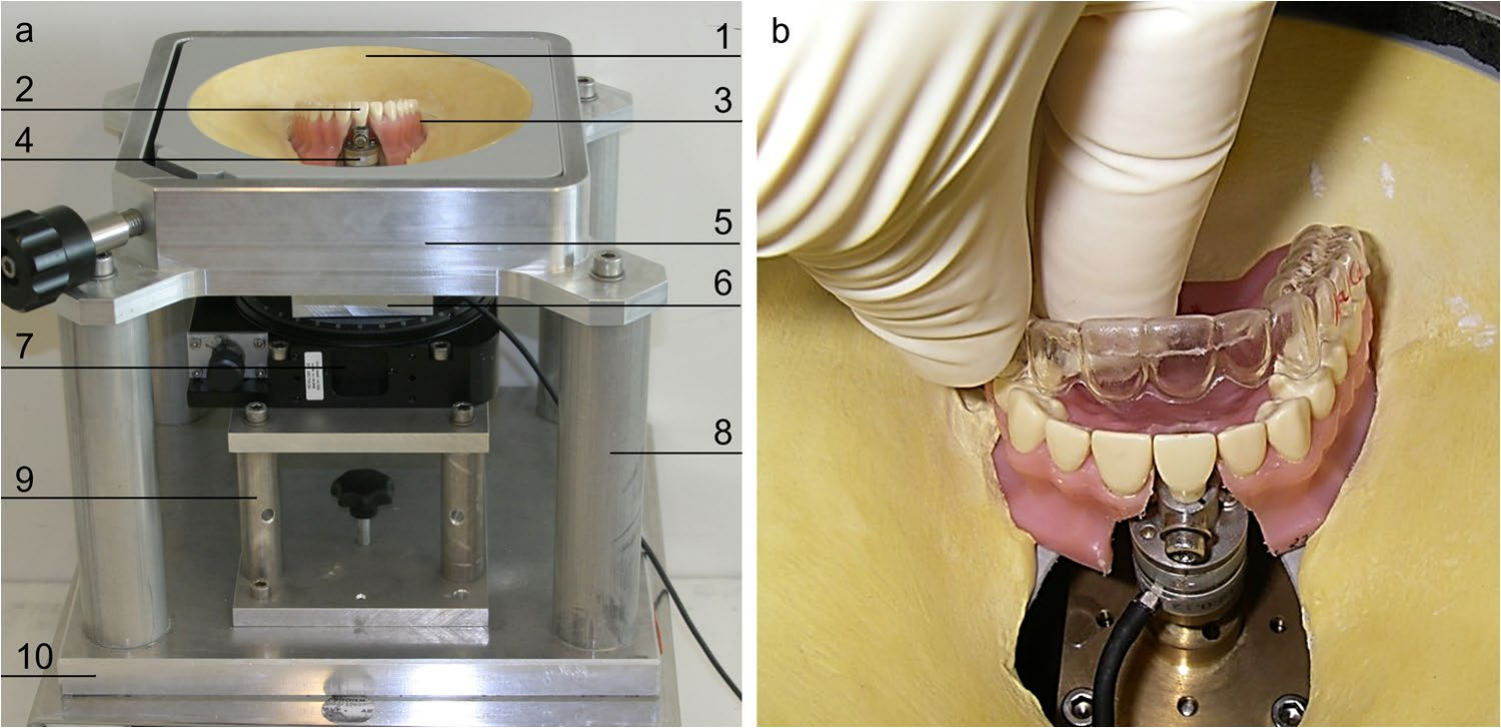
The measuring device and RTA placement. Measurements were performed with a modular measuring device first described by Hahn et al. [14]. **a** shows the overall view of the measuring apparatus with 1: a plastic shell, 2: a separated measuring tooth, 3: a standardized plastic model (Frasaco GmbH, Tettnang, Germany) fixed with plaster, 4: a sensor, 5: a square frame, 6: a sensor base plate, 7: a manual positioning system for precise movement of the measuring tooth, 8: a fixing post, 9: a sensor unit base frame, and 10: a base plate. **b** illustrates the placement of the RTA on the Resind model. A reproducible position of the measuring tooth was ensured by using a plaster key

**Fig. 2:**
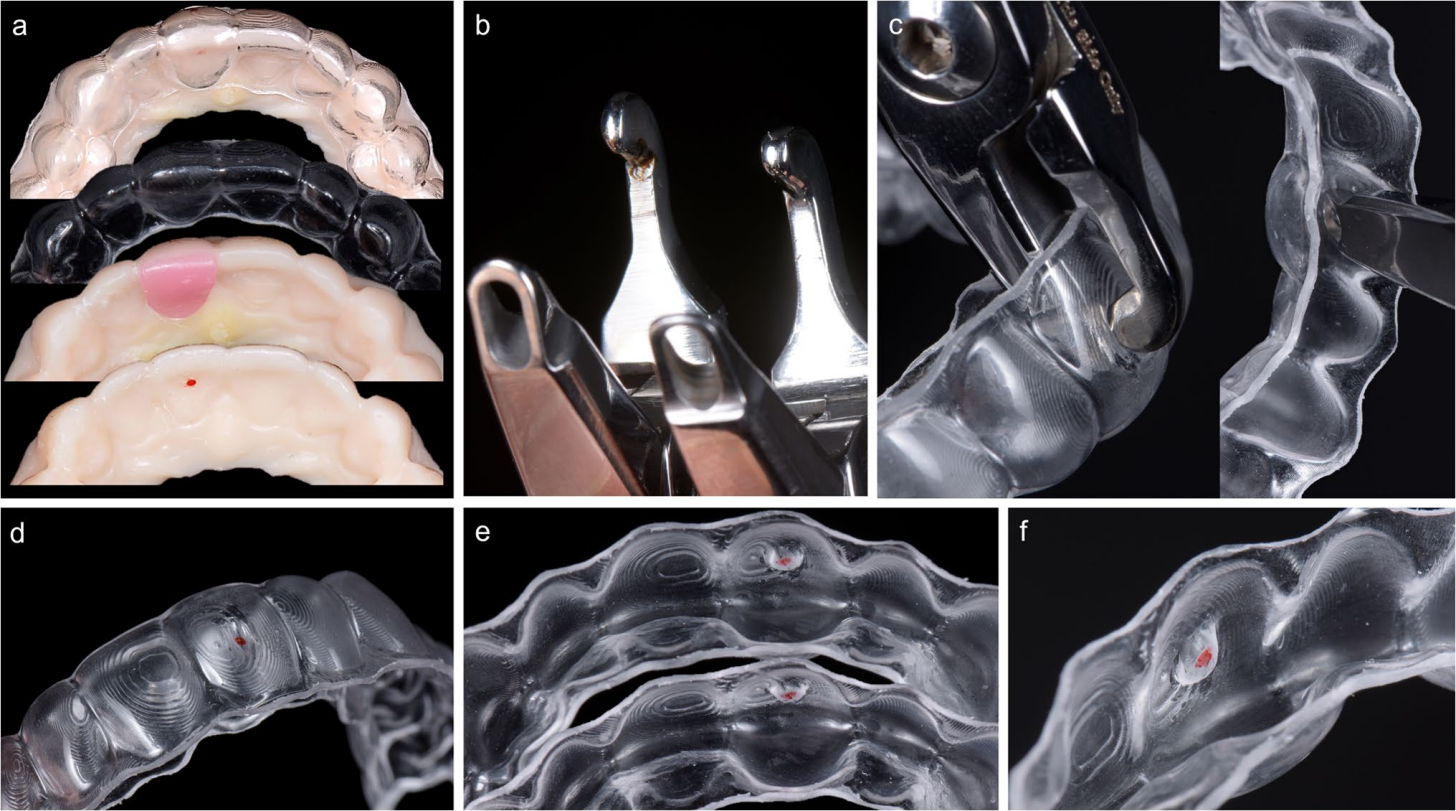
Workflow illustration using Hilliard precision thermopliers. **a** shows the region of force application mirrored from the vestibular surface at 4.55 mm from the incisal edge of tooth 11. Applied thermostable wax (pink) serves spacing for desired palatal tooth tipping, over which a 1-mm PET-G foil is thermoformed. **b** shows the thin and thick precision thermopliers on the left and right sides. **c** shows bump activation of PET-G foil at the desired force application area with 90° activation. **d** shows PET-G foil with a generated bump from the vestibule. **e** shows the bump dimension with the same activation using thick (top) and thin (bottom) precision thermopliers. **f** shows the generated bump using thin precision thermoplier in detail

Force measurements were carried out using a measuring device (Fig. 1) containing a Nano 17 sensor (ATI Industrial Automation, Apex, NC, USA) [14–16, 20, 21]. The sensor was connected to a separate central upper incisor of a standardized resin model (Frasaco GmbH, Tettnang, Germany). Measurements were conducted in a drying chamber. The sensor was calibrated following manufacturer specifications with 1% full-scale accuracy. A resin model with the measuring tooth placed on the measuring device was used (Tetrachrom®, Kanidenta, Herford, Germany) for appliance preparation. Subsequently, a plaster model with a 20-mm-height was assembled (GC Fujirock® EP, GC GERMANY GmbH, Munich, Germany). Two equal plaster copies were produced using Adisil® blau 9:1 (SILADENT Dr. Böhme and Schöps GmbH, Goslar, Germany). The space needed for tooth movement was subsequently created by blocking out the cast with thermostable wax placed nearly up to the incisal edge to avoid friction in this area [19]. Wax was applied on the palatal surface of the incisor and to the vestibular surface of the measuring tooth for vestibular movement. A total of five plaster copies were produced from both casts, as described above, and ten RTAs (five for vestibular movement and five for palatal movement) with an equal extension of 2.5 mm beyond the gingival margin were also produced. Aligners were produced from the Ideal Clear® material (PET-G) (Dentsply GAC, Gräfelfing, Germany) at a 1-mm thickness, as recommended by Hilliard et al. [18], using the “Vacuum Forming Machine” 202 (Dentsply GAC, Gräfelfing, Germany). Two different thermopliers (thin/thick) (Dentsply GAC, Gräfelfing, Germany) were used for spot-thermoforming, as described in the literature [18, 19]. To generate a force component that can be used for tipping, the bumps were positioned in the centre of the palatal and vestibular surfaces of the tooth measured, which was exactly 4.55 mm from the incisal edge of the measuring tooth (Fig. 2a). To ensure bump depths were reproducible, the thermopliers were set to zero before bumps were formed. Therefore, the aligner was grasped tightly with a thermoplier at the point marked for the bump to be pressed in (Fig. 2c). This position was fixed with a hexagon socket screw set, which was placed at the handle of the plier. The screw was turned back before every step of the bump-forming process to thermoform bumps of different predefined depth sizes. The bumps were progressively deepened on the same RTA under investigation every 15° (from 15° to 150°) and adjusted with a modified socket head wrench linked to a goniometer. All measured values were used in the regression analysis. The values at 30°, 60° and 90° were used to compare the force components measured for different movement directions. Additionally, the thickness of the aligner at the bump position was measured with an electronic sliding calliper before and after subsequent activation to correlate the activation degrees with a specific bump height (Table 1). Measurements were made as follows. Before starting the first measuring cycle, the aligner was placed onto the tooth row, and the forces were set to zero. The aligner was removed, and a bump was formed at the intended position. The thermoplier was activated at 30° and warmed up to 85° (using the recommended APT Burner and the HAK < 0 digital thermometer (Dentsply GAC, Gräfelfing, Germany)). When the bump was formed, the inner surface of the thermoplastic appliance was moistened with artificial saliva (Artificial saliva, University-Pharmacy, Goettingen, Germany). Then, the aligner was placed back on the tooth row. Subsequently, the tipping (Fx) and intrusion (Fz) force components of the aligner were measured, whereby the measuring tooth remained firmly in the measuring unit and did not tip in the direction of movement. Moreover, the bump was heightened by activating the respective thermoplier up to 60° and finally to up to 90°. Measurements were made in the same way. The data were recorded five times after each activation step.

**Table 1:** Ratio of bump depths in millimetres (mm) to bump depths in degrees (°) depending on the direction of movement (pal./vest.), thermoplier (thick/thin) and bump depth (30°, 60° or 90°)

Tipping	Thermoplier	Activation	Aligner 1	Aligner 2	Aligner 3	Aligner 4	Aligner 5	Mean
pal	Thick	30°	0.04 mm	0.03 mm	0.02 mm	0.03 mm	0.02 mm	0.028 mm
pal	Thick	60°	0.07 mm	0.07 mm	0.06 mm	0.06 mm	0.05 mm	0.062 mm
pal	Thick	90°	0.1 mm	0.1 mm	0.09 mm	0.1 mm	0.09 mm	0.096 mm
pal	Thin	30°	0.02 mm	0.03 mm	0.02 mm	0.02 mm	0.02 mm	0.022 mm
pal	Thin	60°	0.07 mm	0.08 mm	0.06 mm	0.06 mm	0.05 mm	0.064 mm
pal	Thin	90°	0.12 mm	0.12 mm	0.1 mm	0.12 mm	0.1 mm	0.112 mm
vest	Thick	30°	0.05 mm	0.06 mm	0.05 mm	0.05 mm	0.06 mm	0.054 mm
vest	Thick	60°	0.11 mm	0.1 mm	0.1 mm	0.09 mm	0.1 mm	0.1 mm
vest	Thick	90°	0.15 mm	0.14 mm	0.14 mm	0.14 mm	0.14 mm	0.142 mm
vest	Thin	30°	0.04 mm	0.05 mm	0.04 mm	0.04 mm	0.05 mm	0.044 mm
vest	Thin	60°	0.08 mm	0.09 mm	0.09 mm	0.09 mm	0.1 mm	0.09 mm
vest	Thin	90°	0.13 mm	0.15 mm	0.13 mm	0.13 mm	0.16 mm	0.14 mm

### Statistical analysis

Data were analysed using multifactorial univariate analysis of variance (ANOVA). Due to the small sample size, a compound symmetry structure of a covariance matrix was assumed. For further analysis, the mean value of the repeated measures was used as a dependent variable. All analyses were performed using SAS® software (SAS Institute Inc., Cary, North Carolina USA) at a significance level of *α* = 5%.

## Results

**Fig. 3:**
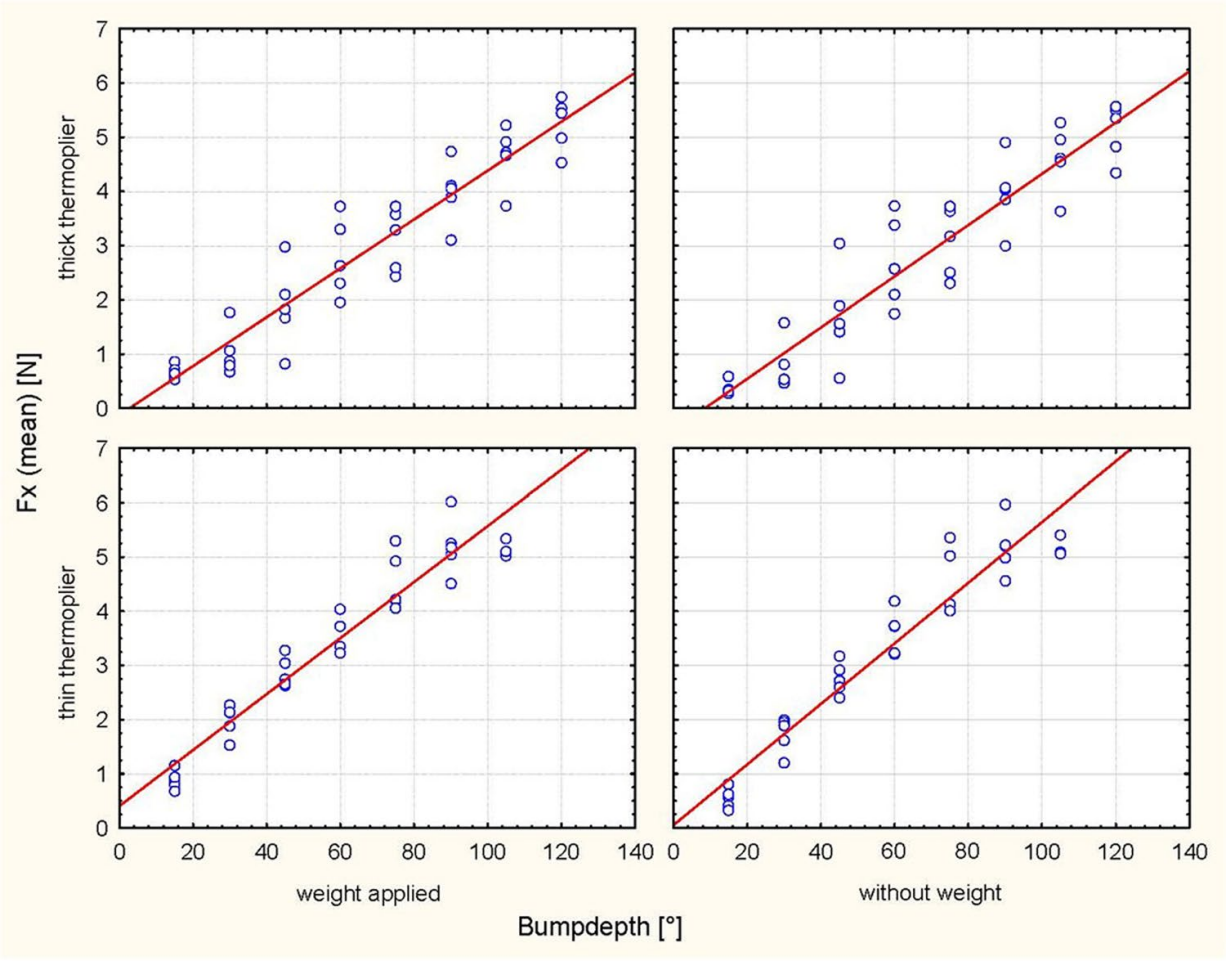
Scatterplot of the regression analysis (Fx, palat. tip). Plot of the horizontal forces (Fx) during palatal tipping as a function of the respective bump depth, the thermoplier used (thick and thin) and the weight applied (with and without)

**Fig. 4:**
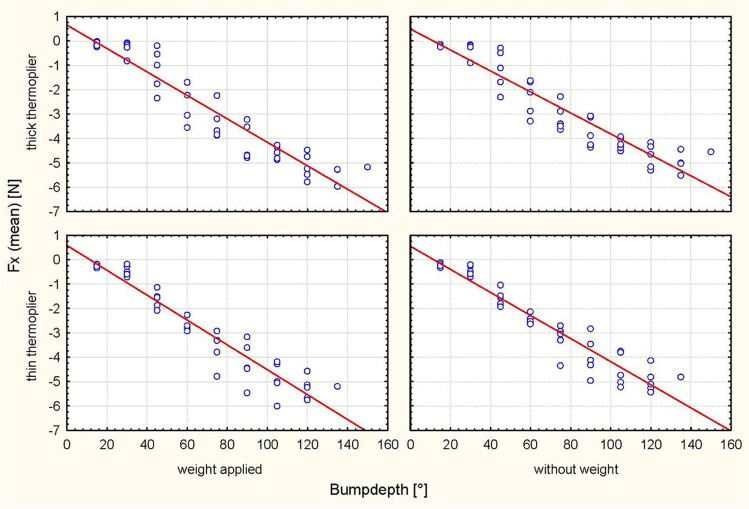
Scatterplot of the regression analysis (Fx, vest. tip). Plot of the horizontal forces (Fx) during vestibular tipping as a function of the respective bump depth, the thermoplier used (thick and thin), and the weight applied (with and without)

**Fig. 5:**
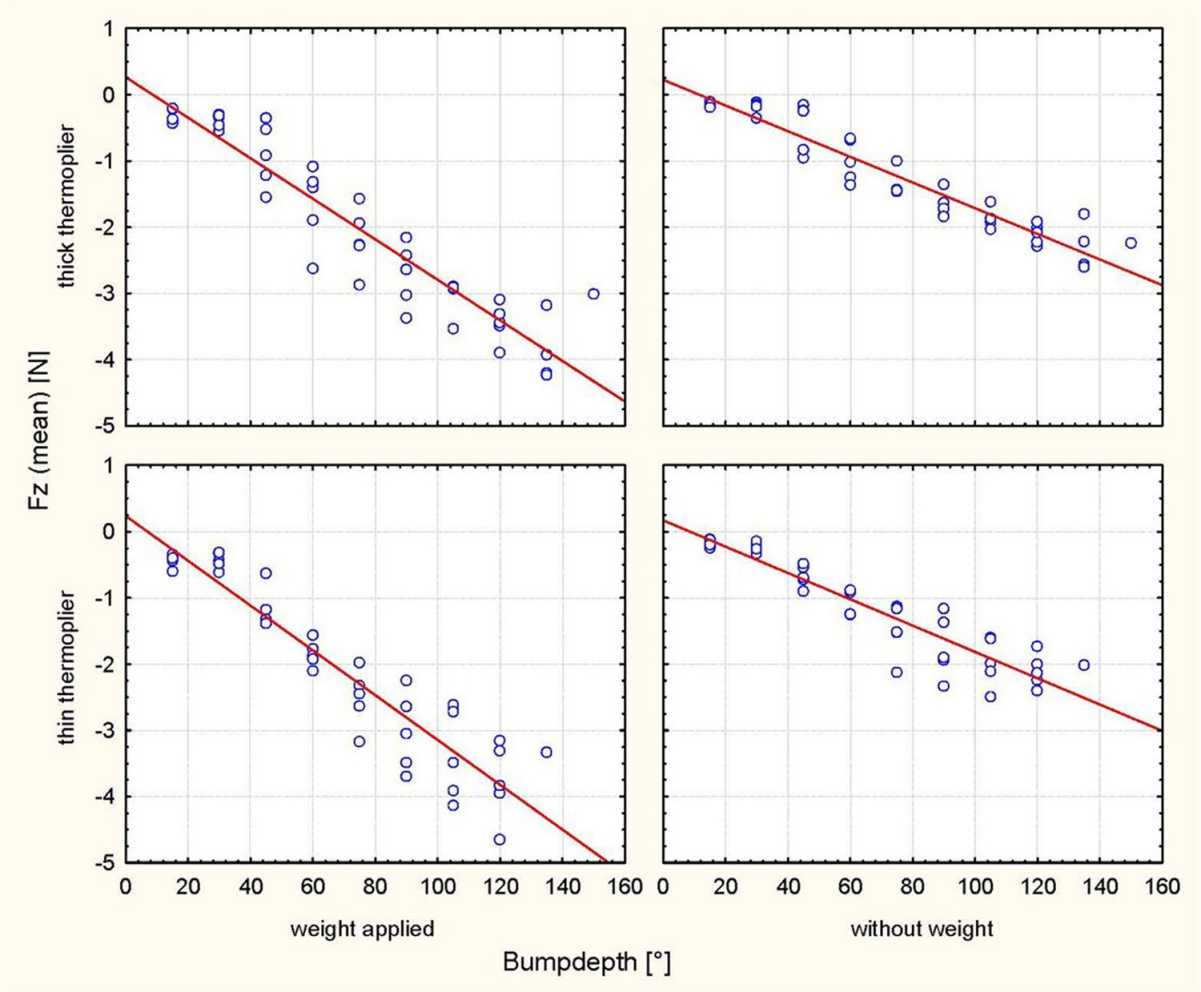
Scatterplot of the regression analysis (Fz, palat. tip). Plot of the intrusive forces (Fz) during palatal tipping as a function of the respective bump depth, the thermoplier used (thick and thin) and the weight applied (with and without)

**Fig. 6:**
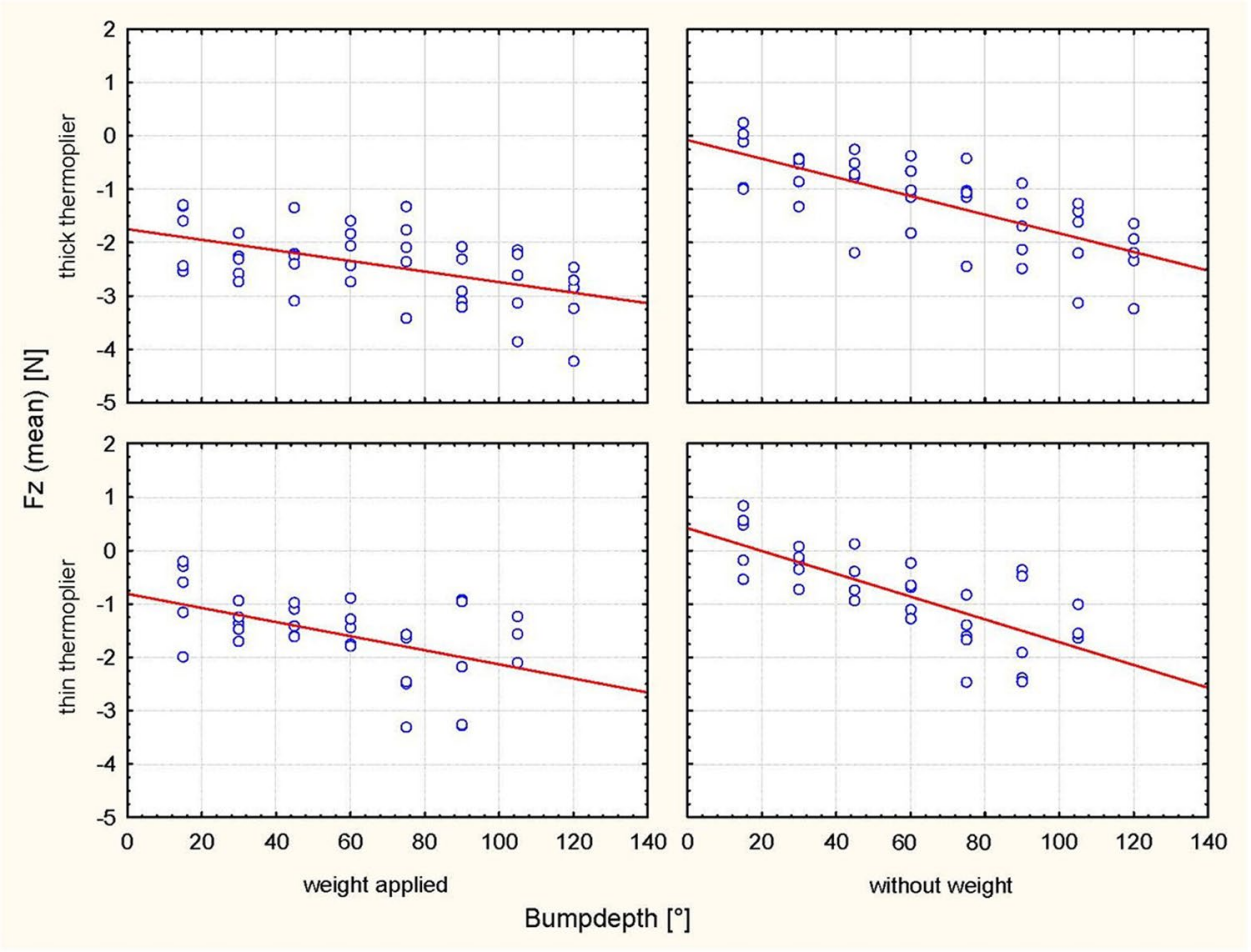
Scatterplot of the regression analysis (Fz, vest. tip). Plot of the intrusive forces (Fz) during vestibular tipping as a function of the respective bump depth, the thermoplier used (thick and thin) and the weight applied (with and without)

Activation at 30°, 60° and 90° resulted in different bump heights depending on the tipping direction and depending on the thermoplier (thin/thick) (Table 1). The analyses revealed significantly higher values of the Fx and Fz force components with increasing bump height for every step of activation in all cases (*P* < 0.0001) (Table 2; Figs. 3, 4, 5, 6). Tables 3 and 4 summarize the mean values, standard deviations (SDs) and the minimal and maximal force values for Fx and Fz for each step of activation (30°, 60° and 90°, respectively). Table 3 also lists an example of the resulting moment of force for a crown length of 9.1 mm, which would arise based on the results of Sia et al. [24] compared to the present setup under clinical conditions with an eccentric force application of 8.63 mm on average to the centre of resistance with a tooth length of 27.2 mm and a root length of 18.1 mm. Different horizontal and vertical forces were generated depending on the position of the bump (palatal/vestibular), despite identical bump depths. The horizontal forces exerted stronger effects on palatal tipping. However, this result was statistically significant only for the bumps applied for palatal tipping with the thin thermoplier (Table 5). The force component Fz exhibited the lowest values during vestibular tipping at 60° of activation by using the thick thermoplier (0.1 mm bump depth) without occlusal load (− 0.1 N (SD 0.32)). The highest Fx force values were obtained at 90° activation (0.112 mm) during palatal tipping using a thin thermoplier with occlusal load (5.20 N (SD 0.54)). In general, Fx values were higher than Fz values. In detail, the Fx force values ranged from − 0.3 N (SD 0.3) to 5.20 N (SD 0.54), where sign indicates the tipping direction (− = vestibular/intrusive direction, +  = palatal direction)) (Tables 3 and 4).

**Table 2:** ANOVA for force direction and degree of activation, in consideration of weight, thermoplier, bump depth and their interaction with each other

Effect	*p*-value
Horizontal force (Fx) during vestibular tipping	
Weight	0.3811
Thermoplier	0.4606
Weight × thermoplier	0.9968
Bump depth	** < 0.0001**
Weight × bump depth	0.4225
Thermoplier × bump depth	0.9637
Weight × thermoplier × bump depth	0.8992
Horizontal force (Fx) during palatal tipping	
Weight	0.5884
Thermoplier	** < 0.0001**
Weight × thermoplier	0.9553
Bump depth	** < 0.0001**
Weight × bump depth	0.6064
Thermoplier × bump depth	0.4080
Weight × thermoplier × bump depth	0.9814
Intrusive force (Fz) during vestibular tipping	
Weight	** < 0.0001**
Thermoplier	0.3212
Weight × thermoplier	0.6658
Bump depth	** < 0.0001**
Weight × bump depth	** < 0.0001**
Thermoplier × bump depth	0.6850
Weight × thermoplier × bump depth	0.7981
Intrusive force (Fz) during palatal tipping	
Weight	**0.0002**
Thermoplier	**0.0261**
Weight × thermoplier	0.2711
Bump depth	** < 0.0001**
Weight × bump depth	0.1402
Thermoplier × bump depth	0.4642
Weight × thermoplier × bump depth	0.9799

Significant *P*-values are presented in bold font

**Table 3:** Means and standard deviations of horizontal forces (Fx) for bump depths of 30°, 60° and 90° with palatal and vestibular tipping, creating the bump using thick and thin thermopliers with and without applied weight (*N* number of aligners, *Var.* variable, *SD* standard deviation in Newton). Resulting force moments were calculated referring to results of Sia et al. [24]

Tipping	Thermoplier	Activation	Weight	Number	Force	Mean (*N*)	SD (*N*)	Resulting moment of force (*N* mm)
vest	Thick	30°	Without	5	Fx	− 0.34	0.31	2.93
vest	Thick	30°	With	5	Fx	− 0.30	0.30	2.59
vest	Thin	30°	Without	5	Fx	− 0.45	0.21	3.88
vest	Thin	30°	With	5	Fx	− 0.46	0.22	3.97
pal	Thick	30°	Without	5	F_x_	0.78	0.47	6.73
pal	Thick	30°	With	5	Fx	1.03	0.43	8.88
pal	Thin	30°	Without	5	F_x_	1.73	0.32	14.93
pal	Thin	30°	With	5	F_x_	1.99	0.29	17.17
vest	Thick	60°	Without	5	Fx	− 2.32	0.74	20.02
vest	Thick	60°	With	5	Fx	− 2.46	0.83	21.23
vest	Thin	60°	Without	5	Fx	− 2.47	0.2	21.31
vest	Thin	60°	With	5	Fx	− 2.7	0.25	23.30
pal	Thick	60°	Without	5	Fx	2.7	0.83	23.30
pal	Thick	60°	With	5	Fx	2.78	0.72	23.99
pal	Thin	60°	Without	5	Fx	3.62	0.41	31.24
pal	Thin	60°	With	5	Fx	3.61	0.33	31.15
vest	Thick	90°	Without	5	Fx	− 3.75	0.61	32.36
vest	Thick	90°	With	5	Fx	− 4.19	0.75	36.16
vest	Thin	90°	Without	5	Fx	− 3.94	0.82	34.00
vest	Thin	90°	With	5	Fx	− 4.24	0.89	36.60
pal	Thick	90°	Without	5	Fx	3.97	0.68	34.26
pal	Thick	90°	With	5	Fx	3.98	0.59	34.35
pal	Thin	90°	Without	5	Fx	5.19	0.51	44.79
pal	Thin	90°	With	5	Fx	5.20	0.54	44.88

**Table 4:** Means and standard deviations of intrusive forces (Fz) for bump depths of 30°, 60° and 90° with palatal and vestibular tipping, creating the bump using thick and thin thermopliers with and without applied weight (*N* number of aligners, *Var.* variable, *SD* standard deviation in Newton)

Tipping	Thermoplier	Activation	Weight	Number	Force	Mean (*N*)	SD (*N*)
vest	Thick	30°	Without	5	Fz	− 0.18	0.1
vest	Thick	30°	With	5	Fz	− 0.39	0.11
vest	Thin	30°	Without	5	Fz	− 0.22	0.08
vest	Thin	30°	With	5	Fz	− 0.44	0.12
pal	Thick	30°	Without	5	Fz	− 0.72	0.38
pal	Thick	30°	With	5	Fz	− 2.34	0.35
pal	Thin	30°	Without	5	Fz	− 0.26	0.30
pal	Thin	30°	With	5	Fz	− 1.34	0.28
vest	Thick	60°	Without	5	Fz	− 0.1	0.32
vest	Thick	60°	With	5	Fz	− 1.67	0.61
vest	Thin	60°	Without	5	Fz	− 1.11	0.19
vest	Thin	60°	With	5	Fz	− 1.84	0.2
pal	Thick	60°	Without	5	Fz	− 1	0.55
pal	Thick	60°	With	5	Fz	− 2.13	0.46
pal	Thin	60°	Without	5	Fz	− 0.79	0.41
pal	Thin	60°	With	5	Fz	− 1.43	0.37
vest	Thick	90°	Without	5	Fz	− 1.68	0.2
vest	Thick	90°	With	5	Fz	− 2.72	0.48
vest	Thin	90°	Without	5	Fz	− 1.74	0.47
vest	Thin	90°	With	5	Fz	− 3.02	0.59
pal	Thick	90°	Without	5	Fz	− 1.69	0.64
pal	Thick	90°	With	5	Fz	− 2.72	0.5
pal	Thin	90°	Without	5	Fz	− 1.52	1.02
pal	Thin	90°	With	5	Fz	− 2.12	1.17

**Table 5:** ANOVA for the respective tipping direction, considering the thermoplier used

Effect	*p*-value
Horizontal force (Fx) following thick thermoplier activation	
Weight	0.5315
Bump depth	** < 0.0001**
Weight × bump depth	0.8960
Tipping	0.1849
Tipping × weight	0.8859
Tipping × bump depth	0.1545
Tipping × weight × bump depth	0.4637
Horizontal force (Fx) following thin thermoplier activation	
Weight	0.3736
Bump depth	** < 0.0001**
Weight × bump depth	0.9871
Tipping	** < 0.0001**
Tipping × weight	0.7695
Tipping × bump depth	0.3310
Tipping × weight × bump depth	0.5487
Intrusive force (Fz) following thick thermoplier activation	
Weight	** < 0.0001**
Bump depth	** < 0.0001**
Weight × bump depth	0.7275
Tipping	**0.0061**
Tipping × weight	0.0649
Tipping × bump depth	** < 0.0001**
Tipping × weight × bump depth	**0.0030**
Intrusive force (Fz) following thin thermoplier activation	
Weight	**0.0009**
Bump depth	** < 0.0001**
Weight × bump depth	0.4870
Tipping	0.4395
Tipping × weight	0.9638
Tipping × bump depth	**0.0010**
Tipping × weight × bump depth	**0.0301**

Significant *P*-values are presented in bold font

### Horizontal force component (Fx)

**Fig. 7:**
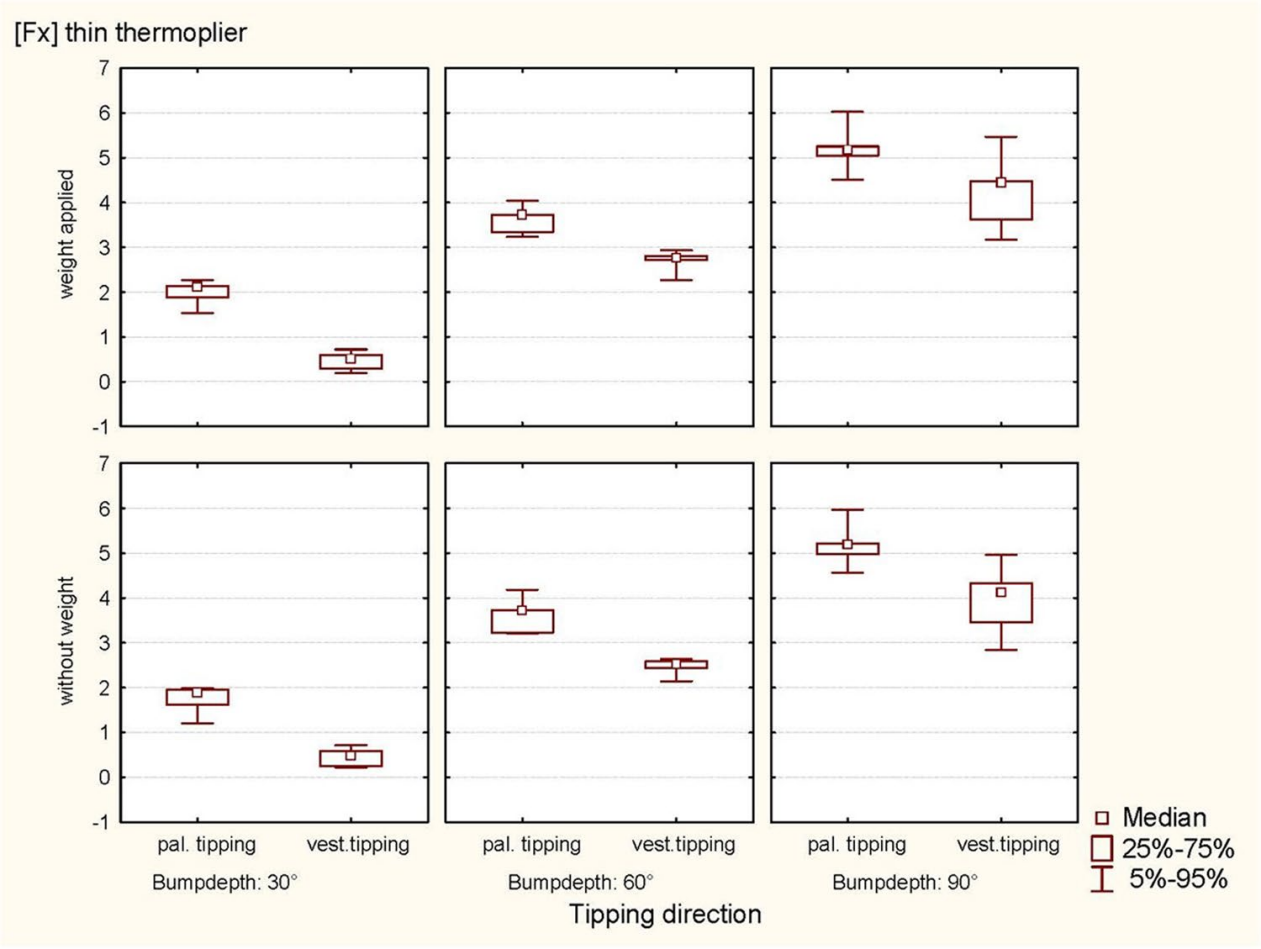
Representation of the horizontal forces (Fx, thin thermoplier). With and without applied weight concerning tipping direction (pal. tipping/vest. tipping) and the bump height (30°, 60° and 90°) using a thin thermoplier

**Fig. 8:**
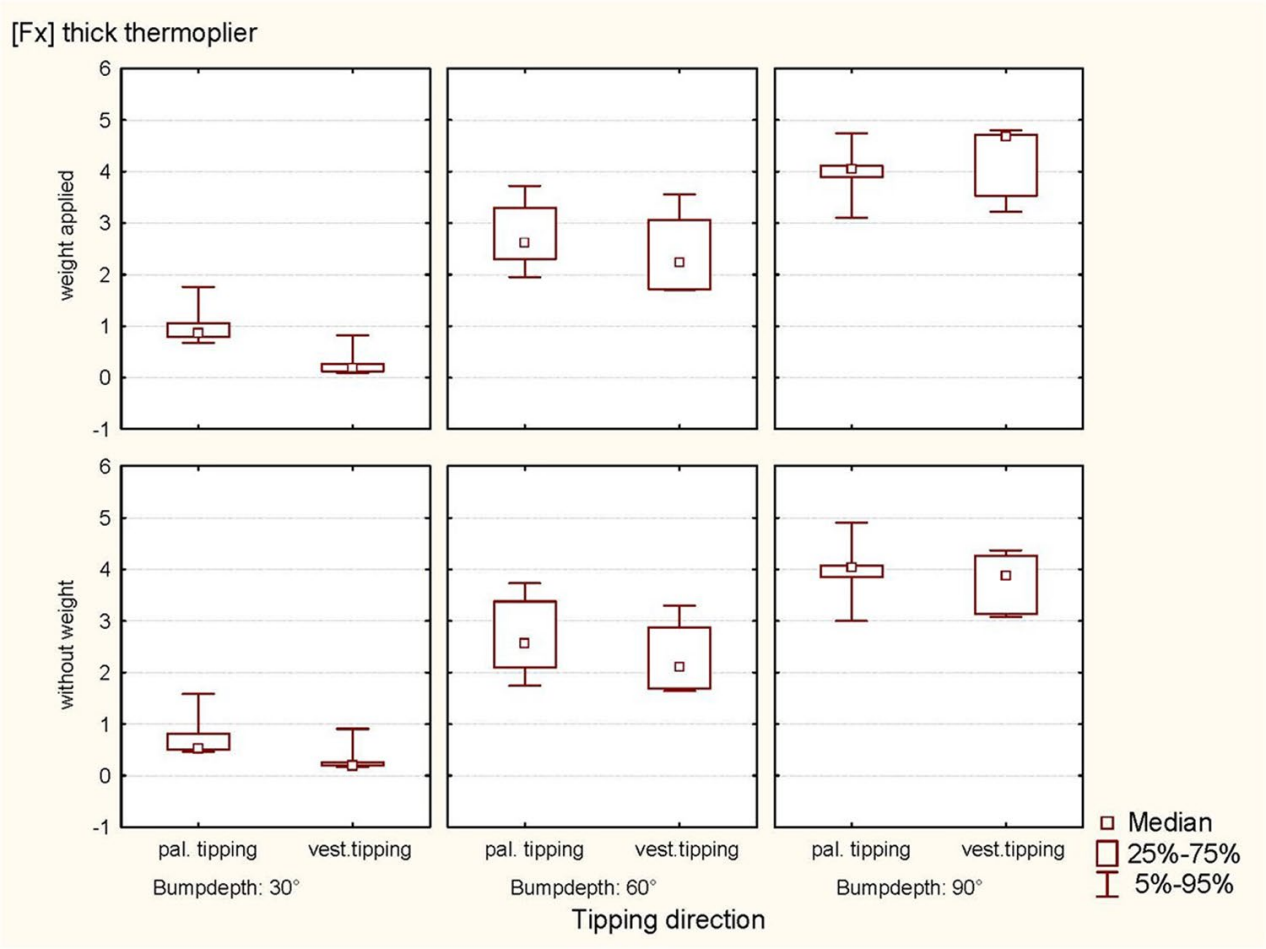
Representation of the horizontal forces (Fx, thick thermoplier). With and without applied weight concerning tipping direction (pal. tipping/vest. tipping) and the bump height (30°, 60° and 90°) using a thick thermoplier

ANOVA revealed a significant influence of bump height on the horizontal force component (Fx) (*p* < 0.0001) during vestibular tipping. In addition, the thin thermoplier (*p* < 0.0001) and the palatal tipping direction (*p* < 0.0001) significantly affected the resulting force Fx. No significant effect of weight (*p* = 0.3811; 0.5884) was observed (Table 2; Figs. 7 and 8).

### Vertical force component (Fz)

**Fig. 9:**
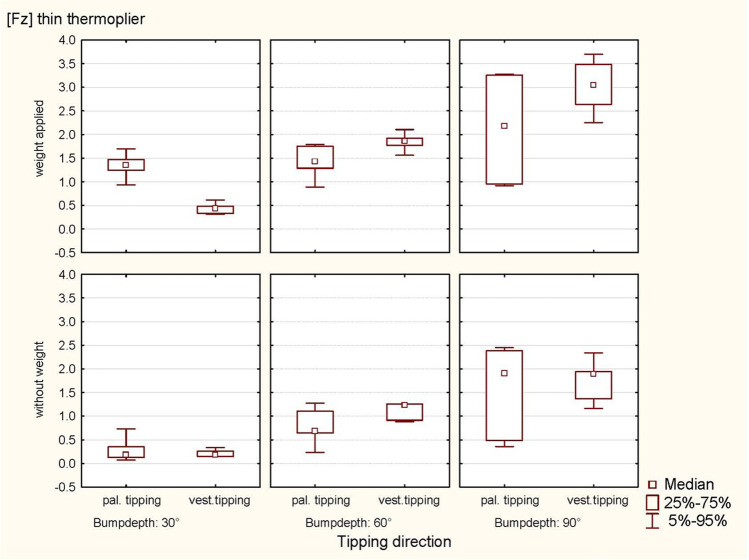
Representation of the intrusive forces (Fz, thin thermoplier). With and without applied weight concerning tipping direction (pal. tipping/vest. tipping) and the bump height (30°, 60° and 90°) using a thin thermoplier

**Fig. 10:**
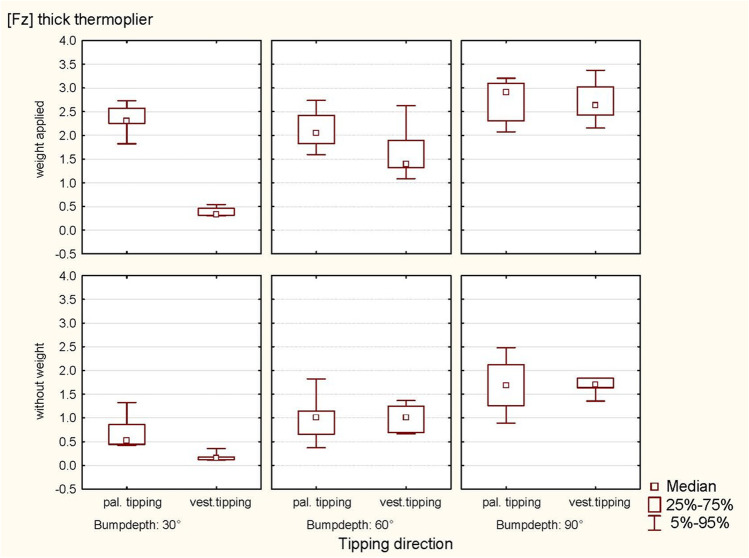
Representation of the intrusive forces (Fz, thick thermoplier). With and without applied weight concerning tipping direction (pal. tipping/vest. tipping) and the bump height (30°, 60° and 90°) using a thick thermoplier

A significant quantitative interaction between weight and bump height was recorded during vestibular tipping (*p* < 0.0001) once the effect of the bump height was recorded in the same direction during the analysis with or without weight (Table 2). A comprehensive subanalysis (splitting the data set by weight) was not performed due to the small sample size. A subanalysis was performed only as part of a sensitivity analysis (with and without weight), revealing a significant influence of bump height (*p* < 0.0001, respectively) (Tables 6 and 7). The simulated occlusal load in the sense of swallowing force generated significantly larger intrusive forces for palatal tipping. The graphical representation, however, showed that the horizontal and intrusive forces also tended to be larger by weight for vestibular tipping, and the horizontal forces tended to be larger for palatal tipping (Figs. 9 and 10).

**Table 6:** ANOVA for relationships of the thermoplier type and the tipping direction

Effect	*p*-value
Horizontal force (Fx) following thick thermoplier activation	
Weight	0.5315
Bump depth	** < 0.0001**
Weight × bump depth	0.8960
Tipping	0.1849
Tipping × weight	0.8859
Tipping × bump depth	0.1545
Tipping × weight × bump depth	0.4637
Horizontal force (Fx) following thin thermoplier activation	
Weight	0.3736
Bump depth	** < 0.0001**
Weight × bump depth	0.9871
Tipping	** < 0.0001**
Tipping × weight	0.7695
Tipping × bump depth	0.3310
Tipping × weight × bump depth	0.5487
Intrusive force (Fz) following thick thermoplier activation	
Weight	** < 0.0001**
Bump depth	** < 0.0001**
Weight × bump depth	0.7275
Tipping	**0.0061**
Tipping × weight	0.0649
Tipping × bump depth	** < 0.0001**
Tipping × weight × bump depth	**0.0030**
Intrusive force (Fz) following thin thermoplier activation	
Weight	**0.0009**
Bump depth	** < 0.0001**
Weight × bump depth	0.4870
Tipping	0.4395
Tipping × weight	0.9638
Tipping × bump depth	**0.0010**
Tipping × weight × bump depth	**0.0301**

Significant *P*-values are presented in bold font

**Table 7:** ANOVA for the influence of weight and thermoplier type

Effect	*p*-value
On intrusive force (Fz) following thick-thermoplier activation with weight applied	
Bump depth	** < 0.0001**
Tipping	**0.0046**
Bump depth × tipping	** < 0.0001**
On intrusive force (Fz) following thick-thermoplier activation without weight applied	
Bump depth	** < 0.0001**
Tipping	0.4558
Bump depth × tipping	**0.0245**
On intrusive force (Fz) following thin-thermoplier activation with weight applied	
Bump depth	** < 0.0001**
Tipping	0.6218
Bump depth × tipping	**0.0016**
On intrusive force (Fz) following thick-thermoplier activation without weight applied	
Bump depth	** < 0.0001**
Tipping	0.5579
Bump depth × tipping	0.4893

Significant *P*-values are presented in bold font

## Discussion

In this investigation, the force delivery of RTAs, modified by different sized spot-thermoformed protuberances (heights from 0.022 to 0.142 mm), was evaluated for tipping an upper central incisor in the vestibular and palatal directions.

The measuring device used was comparable to those previously described in the literature [14, 21]. However, stimulation of the periodontal ligament (PDL) and tooth movement occurring after loading in vivo were not possible. This prevented the results from being relevant for initial force appearance immediately after loading when, due to the viscoelastic PDL properties, no rapid tooth movement can be expected [25, 26]. Of note, a clear concept to relate the force system to tooth movement and the reaction of the different PDL parts has yet to be presented [27, 28]. Nonetheless, the presented load deflection characteristics can provide an approximate potential of force decay in relation to the distance moved by the tooth after loading.

The measurements revealed that force values of 0.35 to 0.60 N for tipping an upper central incisor, as recommended in the literature [29], were reached by a mean bump height of 0.041 mm. This activation refers to an activation approximately 24 times lower than the least recommended height (1 mm) by Sheridan et al. [19]. Furthermore, Sheridan and Hilliard described two essential methods of aligner-guided tooth movement. In addition to bump generation on the active side and blocking out on the passive side, as done in this study, the authors described the possibility of grinding out a malocclusion model by the desired amount on the active side and blocking it out by the same amount on the passive side before thermoforming, thus eliminating the need for active modification with pliers [19]. The authors concluded the same biomechanical effectiveness for both techniques, but this could not be confirmed with the results of the present study, since the smallest activation of 30° in the present study alone was associated with a change in force of 0.1 N because one bump was narrower than the other. This finding seems significant with regard to the modification possibilities of the currently established fitting discrepancy method, since bumps, such as power ridges, are also used here to amplify complex torque movements [22]. Although tooth movement in this case is dictated by the fitting discrepancy and does not occur in free space, it experiences an additional force with additional torque due to the use of a bump, and the smallest changes in bump design can alter the resulting force, which must be carefully dosed, especially for root movements. Consequently, the dimensioning of the bumps or power ridges must be taken into account in the current aligner technology to avoid exceeding the physiological limits of tooth movement. Although the majority of the current literature reports lower root resorptions under aligner therapy [30], special modifications, such as bumps or power ridges, are almost not differentiated in these analyses [31, 32]. However, it is a fact that the root resorptions observed in aligner therapy are generally concentrated in the maxillary incisors [33], i.e., where additional forces for complex movements are most frequently needed. The results of the present study show that considerable force peaks can be generated precisely by simple material exertion and exertion designs.

Another aspect is that studies largely reference established aligner systems whose material composition, attachment designs and bump dimensions are largely kept secret. Although there are currently numerous opportunities for orthodontists to design and manufacture aligners independently, there is still a lack of knowledge about force sizes and the necessary bump designs, which makes independent aligner therapy in this context considerably more difficult in terms of force dosage [31]. The results of this study also suggest that further independent research is needed for a better understanding of force ratios in the fitting discrepancy technique with additional bump modification.

Furthermore, the present study results draw attention to the increased force ratios of aligners under chewing pressure. Although there are numerous chewing simulation [34] and wearing time [35] studies in the current literature, they are primarily devoted to the accuracy of fit [35, 36], the resilience and the general reaction [37] of the variety of materials and layer thicknesses [34] but not to the effects on the patients’ periodontium. Considering the previously discussed aspects of various additional activations (e.g., power ridges) and the expected increase in force peaks, the aligners provided additional force peaks under chewing load, which suggests a summation effect. Assuming that patients have different levels of muscle activation and chewing behaviour, additional aligner activation may need to be considered more critically.

With regard to the foil dimension of 1 mm used in the present work in accordance with the recommendation of Sheridan and Hilliard [19], it should be noted that it was above the currently common dimension of 0.75 mm. However, in terms of the forces generated, this is relative, as the movements occurred in spaced aligner regions and did not act on the entire tooth as they did in the fitting discrepancy method, which would have meant the generation of higher forces per se [21]. According to current research, thicker aligner materials have a higher dimensional stability over a longer period of time [34]; this stability must be assumed in the approach of Hilliard and Sheridan since the aligners are successively reactivated and are thus subject to higher periods of use.

With regard to bump positioning, a central point of impact was chosen in the present work to obtain a well-reproducible initial situation on the one hand and to obtain a general overview of the forces that arise on the other hand. However, the recommendation about bump positioning in everyday clinical practice should be based precisely on the tooth morphology, the desired tooth movement and the biomechanics required for it.

For example, for vestibular tipping, a palatal bump must be included in the creation of the aligner. In the case of a cervical bump position, the force would act on a more horizontally inclined surface than a bump position near the incisal edge, which would modulate the proportion of intrusive forces. Additionally, when a bump is activated, it is in the nature of things that it hits the tooth surface much more vertically and less flatly than, for example, a comparatively flat application of force as in the fitting discrepancy technique; this angle leads to a relatively high torsion of the entire aligner [16]. If the bump is placed as incisally as possible, this effect is expressed in the comparatively highest force application, which is explained by the simple leverage effect of the remaining aligner material tending to reset. Therefore, in the case of more incisal aligner activations, not only the biomechanical conditions of the force system but also the material-specific restoring behaviour at the border areas should be taken into account, and consequently, the bump dimensions should be kept smaller and less prominent. Furthermore, a bump position near the incisal edge leads to increased Fx and Fz force values and may cause the aligner to lift. If the friction of the appliance is high and the bump is positioned near the incisal edge, a higher intrusive force component can be measured. In contrast, if the friction is low, a bump position at the centre of the crown might produce higher force values. Apart from this, it must be considered that incisal activation is limited by the design of the plier head, and thus, incisal bumps directly to the incisal edge could not be created (Fig. 2c).

However, accounting for the broad correlations of bump dimension, morphology and positioning, a clinically straightforward approach for small malocclusions should also be investigated against the background of Hilliard’s easy-to-use system; this approach would allow possible action during the early retention phase with low material and cost requirements. If, for example, 1-mm-thick PET-G retention appliances are used in clinical use anyway, as is generally accepted, only a small amount of additional work would be required to block out the dental cast by the amount of the desired tooth movement with wax prior to thermoforming or, in the case of an intraoral scan, to block out this area digitally. Subsequently, the retainer material could also be used for thermoforming, and the produced aligner could be used over a longer period of time and reactivated within the range of acceptable orthodontic forces. The results of the present work can be used as an orientation for simple tipping movements with a central point of applied force, considering the limitations of the measurement setup. Furthermore, it could be shown that higher activation levels, as they were originally described, should be avoided in any case, since simple aligner manipulations can generate remarkable forces.

## Conclusion

The investigated therapeutic approach represents an interesting alternative for simple tooth malocclusions due to its uncomplicated clinical implementation and the option of making modifications to RTAs with little effort, especially in view of the currently increasing material costs of aligner therapy. Furthermore, the measured values illustrate the remarkable force capacity of RTAs as well as the increase in force systems by minimal modifications; these factors also need to be considered or investigated in more detail regarding the designs of established procedures.

## Data Availability

All data generated or analysed during this study are included in this published article (and its supplementary information files).
