# Correction to: Force delivery modification of removable thermoplastic appliances using Hilliard precision thermopliers for tipping an upper central incisor

**DOI:** 10.1007/s00784-022-04575-x

**Published:** 2022-06-08

**Authors:** Bernhard Wiechens, Phillipp Brockmeyer, Teresa Erfurth‑Jach, Wolfram Hahn

**Affiliations:** 1 Department of Orthodontics, University Medical Centre Goettingen, University of Goettingen, Robert‑Koch‑Str. 40, 37075 Goettingen, Germany; 2 Department of Oral and Maxillofacial Surgery, University Medical Centre Goettingen, Robert‑Koch‑Str. 40, 37075 Goettingen, Germany; 3 Bremen, Germany; 4 Goettingen, Germany

**Correction to: Clinical Oral Investigations**

**https://doi.org/10.1007/s00784-022-04560-4**

After publication of the original article [1], the authors noted that the information that Author 1 (Bernhard Wiechens) and Author 2 (Phillipp Brockmeyer) have contributed equally to this study was not included.

**Author details**

1 Department of Orthodontics, University Medical Centre Goettingen, University of Goettingen, Robert-Koch-Str. 40, 37075 Goettingen, Germany

2 Department of Oral and Maxillofacial Surgery, University Medical Centre Goettingen, Robert-Koch-Str. 40,37075 Goettingen, Germany

3 Bremen, Germany

4 Goettingen, Germany

* Bernhard Wiechens and Phillipp Brockmeyer have contributed equally to this study.

**Reference**

1. Wiechens B, Brockmeyer P, Erfurth-Jach T, Hahn W. Force delivery modification of removable thermoplastic appliances using Hilliard precision thermopliers for tipping an upper central incisor. Clin Oral Investig. 2022 May 31. https://doi.org/10.1007/s00784-022-04560-4. Epub ahead of print. PMID: 35641836.

The original article has been corrected.

